# Dr. Taylor on Filling Teeth

**Published:** 1850-10

**Authors:** James Taylor


### DENTAL REGISTER OF THE WEST.

VOL. IV. OCTOBER, 1850. NO. 1.

### DR. TAYLOR ON FILLING TEETH.

AN ADDRESS DELIVERED BEFORE THE MISSISSIPPI VALLEY ASSOCIATION OF DENTAL SURGEONS, AT CINCINNATI, SEPTEMBER 10, 1850.

BY JAMES TAYLOR, M.D., D.D.S.

*Gentlemen of the Mississippi Valley Association of Dental Surgeons:*—We convene on the present occasion in accordance with the requisition of that constitution which binds us together as a society. May we not with profit take a retrospect of the past year, and refresh our memories with the many mercies enjoyed. Disease, like the Simoon's blast, has swept over the land. Thousands have fallen. We are preserved. A merciful Providence has kept us, and so far as I know, not one of this association is numbered with the dead. The scourge has passed; health, peace, and plenty abounds; and the dried up streams of social life, which had been blasted and checked during the prevalence of the epidemic, begin again to ebb and flow with their accustomed healthful and benign influence;—and on the tide of this renewed and reviving stream of social life, we are permitted again to assemble and consult on the best interests of our profession.

Let us endeavor to do this with a proper feeling of our individual responsibility, duly impressed with the importance of the profession to which we belong. "Let us press on towards the mark for the prize," each endeavoring to bring into the grand depot of dental knowledge something which shall benefit our race, and give our science a still larger claim on the confidence of an enlightened public.

We meet to compare practice, to receive and impart useful knowledge, to spend a few days in the discussion of scientific subjects, and extend to each other that fraternal feeling which should ever characterize those of the same profession. Let us, therefore, throw aside every feeling but love—love for each other, and for that truth on which the temple of our science must be erected. Let every sentiment, every preconceived opinion be held subservient to this great principle, this broad platform on which all true science must stand. We meet untrammelled by any of the *isms* of the day; dental science being only a speciality of general surgery, stands more securely aloof from the innovations of the age. No Homœopathic dose will suit in the extraction of a tooth—no Hydropathic ablution can insert a set of teeth; and since the late attempt to introduce steam into the practice of dental surgery, no change has taken place in the regular routine of the profession. In the west we have happily escaped even the heat and excitement of the amalgam controversy. I know not if even the southern improvement on the screw has been able to disturb in a single instance the equanimity of any member of this society.

Medical science, in all its branches, constitutes one of the most instructive and ennobling studies in which we can engage. Instructive, because at every step we behold the continued exhibition of infinite wisdom; and as we advance and learn more and more of the mysterious frame work to which this science is applied, we can the more fully enter into the feelings of the psalmist, until we with him can exclaim: "Man is fearfully and wonderfully made."

The study is ennobling, because it teaches us the frailty of man—our utter dependence on a power Supreme. It takes us from the study of nature to nature's God—points us back through the long vista of time, to a period when perfection was stamped on all the work of creation. Man was perfect; disease and death knew not a victim. Imagination can scarce do justice to the picture of man in his perfection. Every movement is ease, and every muscle acts its part in perfect harmony. No blot or blemish is to be seen. The ear catches the most distant sound as its vibration strikes on its delicate and finely attuned tympanum; melodies afar as by angels sung, are caught on the passing zephyrs. The eye pierces the distant horizon and beholds spread out in the vast panorama; a broad and extended domain filled with the beauties of a beneficent Creator. Every sense is attuned and works harmoniously; each organ is in unison, and the teeth present a perfection, but rarely if ever seen. But man fell, and disease and death has ever since made sad havoc on the human race. The most dense and indestructible part of our system is not exempt from this direful calamity; and the teeth with their beautiful enamel; hard and compact in its basalt, like columns, capping and shielding the hardest portion of the osseous structure, also yield to the fearful sentence: "Dust thou art, and to dust thou shalt return." Thus man has been made to feel, that to prolong his days he must employ all the aids which God in his mercy has scattered in rich profusion around him. This feeling of self-preservation has led to an investigation of almost every material substance in nature, until their constituent principles and remedial effect have been discovered.

How delightful the task to mitigate the sufferings of a fellow being, to arrest disease in its rapid progress, and give ease and comfort. This is our privilege, and well does it repay for days, months, and years of labored study.

But, gentlemen, I would not forget that our meetings are designed to be of a strictly practical character—that however pleasant it might be to speculate on the gradual approach and development of disease; and that science which points out a remedy, yet at this time I have another subject assigned me, and hence must forego this pleasure and call your attention at once to the operation of filling teeth. It will be recollected that at the close of our last meeting this subject was under discussion.

I am aware that it is a difficult subject to handle. There are many difficulties which so often occur in the proper performance of this operation, that to anticipate them at all times is almost impossible, and they are even difficult to describe. I therefore do not expect to do the subject justice. I can only hope to in part describe my mode of operating, and elicit a free and full discussion of the subject.

There is no operation in dental surgery so important—none which is capable of affording so much of real service to our patients. The operation of extraction, it is true, is often of vast importance and absolutely essential to the health and comfort of an individual, yet how seldom would this be necessary if the former was resorted to in time and, successfully performed. The operation of filling, steps in as a preventive to extraction, and the perfection of our science would do much to prevent the need of the latter.

The progress of our science points to perfection and this accomplished,—the entire preservation of the dental organs is the result. Hygeanic treatment, so far as applicable, holds of right a high place in our regard; yet with our present knowledge how inadequate this would prove. How could we control our patients in this respect? And without an entire control of diet, and all that pertains to cleanliness, but little could be accomplished. To correct the evils resulting from constitutional defect in the dental organization, we should have to control mothers, nurses, diet, exercise, and indeed many of those infantile diseases which so much baffle the medical practitioner, and consign to an early grave one third of the human race.

But to return to the special object of this address, I would reiterate the remark made at our last meeting when this subject was under discussion, which "was that considering the importance of the operation—its frequency and the difficulties attending it, it is strange that so little of that which is specific in the detail, has been given by those who have written on this subject." It is not so in relation to any of the operations of general surgery; and yet there is no other operation performed one twentieth as often, (save the extraction of teeth.)

It is very easy to say that the decay must be thoroughly removed, that the cavity must be well shaped, and the gold well consolidated. This is all true, and appears so simple and plain that every man thinks he may do it easily, and indeed nothing is more common than for young and inexperienced operators, to think they can fill a tooth as well as any one. I might give repeated examples of this. I recollect one case, where the young gentleman said, that his fillings had only one fault, and that was they were too *hard*.

In the present discussion of this subject, I shall so far as brevity will permit, follow the course which I pursue before the dental class. In doing this, however, I shall not be able to go into all the detail, but will give general principles and classifications. All cavities are not alike either in shape or location, the *modus operandi* also varies; classification is then necessary for description. How can you describe without a name? To do this so as to be well understood, the name should locate the cavity, but to avoid confusion such names should be adopted as would simplify as much as possible; hence I have adopted the following general classification: First, central cavities. These embrace all those situated on the cutting or grinding surface of the teeth—the molars and bicuspides most generally present such cavities.

Second, the labial. These are situated on the outer or labial surface of the teeth. The molares most generally present this location of decay; yet in certain forms of disease it extends to the incisores, canine and bicuspides. I might say a certain form of decay first attacks these, then the molares.

Third, the approximal. These are situated on the approximal surface of the teeth, and form a class as a general rule the most difficult to treat. They are found on all the teeth, and in form are modified by the shape of teeth the affected.

Fourth, the palital. These are located, as the name denotes, on the palital surface of the teeth. They are the most rare of any—occurring most frequently on the lateral incisors and first and second molares above.

These four divisions embrace nearly all, yet we have at times combinations of these. There is the central and approximal combining the central and labial, the central and palital, the palital and approximal in the incisores. Other combinations may occur, but these are the most usual.

Let us consider the four distinct and marked divisions, and in the order named; then, if time permit, consider the combinations, with those presenting an exposed nerve, &c., &c.

First, the central. This class of cavities are most generally found in the molares and bicuspides, and are the most accessible and least difficult to fill of any. Yet even here where success would appear always certain, how often are we are mortified to find that the most plain and apparent principles of mechanism have been neglected. The reason why such fillings often fail and come out, is not because the cavity may not have been well cleansed and formed, but from the mode of introducing the gold. This, I think, will be made apparent as we proceed in this discussion.

The first duty of the dentist is carefully to examine the nature, depth, and extent of the cavity he is about to fill. If the decay is deep, he wishes to know if the nerve is exposed, the condition of the bony structure and the strength of the walls of the cavity. To determine these several conditions, he must carefully probe the decay, scrutinize the color of the enamel and tooth, &c., &c. When this has been satisfactorily done, the next step is to uncover as it were the disease; that is, break down the frail portions of the enamel which generally hide from our view the extent of the decay in the bony structure.

Close observation will enable us to determine pretty accurately the extent of the decay, by the color of the tooth; for the decay beneath is reflected through the enamel, giving it a black, blue, or white appearance, depending on the nature and color of the decay. It is always, however, different from the healthy color of the tooth.

The first step in the preparation of the cavity, is therefore to remove this enamel, and for this purpose I have some half dozen cutting instruments of different forms; flat on one side and beveled on the other; so as to form a cutting edge like a chisel; two pair of these come to a sharp point. For the lower teeth, the cutting part of the instrument is bent at right angles with the bar. In the use of these I commence at the center of the decay, and break in by small piece after piece, the enamel, until I find the border firm and strong. Then with an excavating instrument remove all the softened portion of the tooth, and by the time this is done every point around the opening is so exposed as to indicate the exact line to which we should carry the use of the cutting instrument. I use these instruments because they are more speedy, and produce less pain than the drill. After, however, I have progressed thus far in the operation, if the cavity is round or will bear being thus formed, I select a drill as near the size of the decay as possible, and with this, round the mouth of the cavity; but it must be recollected that all these cavities are not of this shape, nor will admit of this construction. We find on the molares, and more particularly on the inferior small grooves or fissures running at right angles across the center of these teeth, resembling a suture, uniting the four prominences on the grinding surface of the teeth. The center where these fissures cross is depressed, and often so much so as to form a lodging place for small particles of food. Here is the point where disease commences, and it extends along these sutures to where they disappear in the smooth enamel which forms the outer surface of the tooth. At the extremes of these sutures there is often observable a depression much like the central one, and each of these points like the central becomes the seat of disease. When decay has progressed to any considerable extent, all the points of disease are merged in one, forming a large and single cavity; but where this is not the case, we find a condition of things requiring much time and skill to overcome. If the disease has followed the line of these sutures, we will have an irregular shaped cavity, with four points of healthy bone corresponding to the four prominences of the tooth, running in toward the central decay.

These may be too firm and sensitive to bear cutting away. It will not do, however, to drill out the center and fill, leaving a dark line along the extent of these sutures; but these must also be cut out, each forming a cavity of itself sufficient to retain a plug if there was no connection with the center. In the preparation of such cavities for filling, I first open the center with my cutting instruments, then follow these sutures as far as diseased, remove the loose carious portion of tooth with an excavater, and then determine, by careful observation, the form which is best to give the cavity. In some cases, I first with a drill round the center, then with a much smaller one, drill out each suture. This is, however, merely to give form to the border of the cavity; for after this is done, I prefer using suitable excavaters, so bent and pointed that I can reach any point of the cavity. In the use of these instruments, I need hardly say that the cutting should be from the nerve; this is particularly necessary in deep decay. Every practitioner of experience and observation must have observed, the cessation of sensibility to some extent which follows the perfect removal of decay from the sensitive tooth. How shall we account for this: is it the diseased portion already softened by disease, the touching of which gives such pain? If not, why is the sensibility so sensibly diminished when this is removed? To avoid as much pain as possible in the removal of such decay, as well as to facilitate the operation, I select some point of the cavity most accessible, and here with an excavator well adapted to the cavity, reach as soon as practicable the firm bone beneath. Then with each stroke of the instrument, first touch the healthy bone, and drawing outward remove the carious portion. In deep decay this can be done by circling around the nerve, leaving the portion over the nerve to be last removed. This is specially necessary when we anticipate its exposure. When, however, there is much sensibility around the border of cavity in the bone just beneath the enamel; we need not expect the exposure of the nerve. In deep decay when there is no sensibility at this point. I should expect a dead or exposed nerve. There is much on this part of the subject which might be dwelt upon; but a more appropriate time will be when we come to the filling of the teeth over exposed nerves. Enough has been said to give some idea of the different conditions of decay to be met with, particularly in the inferior molares. Sometimes the same is observable in the superior. It is needless to say that these conditions must give form to the cavity in either instance; but in the superior molares we have more frequently two distinct cavities, the anterior large and round. The posterior oblong, situated on a suture which marks off a section of the teeth, sometimes one-fourth, one-third, or more, running from the back part of the posterior or labial prominence of the teeth, obliquely forward toward the center of the palital surface of the tooth, dropping it is true back of the center, lining off as it were the posterior palital prominence of the tooth. We have then in these teeth very frequently, two depressions, each becoming the seat of disease and only separated by a small portion of healthy bone. The suture running from the centre back, is often merged in the other, and when decay has progressed far, uniting both in one cavity. When these two decays are separated by healthy bone, strong and firm, they are to be treated as two separate cavities; but if this be not the case and the decays meet, the intervening bone although but partially destroyed by disease, should be as far as possible removed. This is done best with the cutting instruments.

One or two peculiarities occur in the central decay of the bicuspides, which are worthy of note before we further describe the manner of preparing these cavities for the reception of a plug. We have here but the one suture running across the teeth, and this passes from approximal surface to approximal; but the minor or palital prominence of the tooth being placed central, and the labial also being highest in the center, this suture is sometimes nearly if not quite divided by an elevation which passes from the labial to the palital prominence. Thus forming two depressions. This condition of things is met with not only in the superior, but also at times in the inferior bicuspides. Disease often takes place at both these points, and the necessity of one or two fillings will depend on the extent of decay. This form of teeth giving rise to two depressions, should be borne in mind, for if decay also takes place on the approximal surface, we shall have a compound cavity at times difficult to manage. When I come to speak of approximal cavities, however, I shall have occasion to especially allude to the effect which they exert in the formation of these cavities. All teeth may be affected with cavities on their grinding or cutting surface, forming central cavities; but they require not a separate consideration, as the same treatment would be applicable.

It will be observed from what has been said, that the form of the cavity must often depend on the several conditions already described. The manner of exposing the decay and removing the caries has been alluded to, but the shape of the inner portion of the cavity has not been described, I allude particularly to the walls which bound the cavity. Shall these be perpendicular or shall they be beveling, forming a much larger cavity within than around the margin? Much has been said on this subject, yet I suppose that no one would now contend for an enlargement of the cavity just beneath the margin, forming what is called a neck. The impossibility of properly filling such cavities is acknowledged by every experienced operator. Such a form of cavity, although in certain other locations, *in part* may be admissible and even advantageous, yet in those now under consideration it is always improper. I prefer that the walls of the cavity, (and particularly the central,) be perpendicular—making the cavity as large within as at the opening, but *not* much larger. The disease should be thoroughly removed. I mean by this, that not only the discolored portion of the tooth should be removed, but all that portion which has been acted upon by the decomposing agent inducing decay. But it may be asked how this is to be determined? The nature of the decay must here be duly considered. Caries assume three marked characteristics as it regards color—black, brown, and that called white. In the black, the carious portion is generally hard, and the bone beneath often discolored deeper than the extent to which decomposition has extended. This discolored bone has not always lost its vitality; not knowing, however, but the discolored portion may have been sufficiently acted upon by the agent inducing decay, to ultimately produce decomposition. It is thought best that it should be removed. This practice, although not always absolutely necessary, yet it is considered safer. Still I prefer a cap of hard discolored bone for the protection of a nerve to any artificial one that can be made. I think in this variety of decay we should be governed some by the density and sensibility of the bone as it regards the amouut to be removed. Solid, well-constituted and healthy bone is as good if not better than any filling which can be introduced, and should never be removed except where it is necessary to give shape to the cavity.

In the brown variety, we have a more softened condition of decay, the dentine less discolored, the line of demarkation between the diseased and healthy portion more distinct, the earthy portion appears to be removed, leaving the cartilaginous, which still adheres to the living structure, and indeed seems as yet not to have lost its own vitality. This decay is more rapid in its progress than the former, yet the portion of the bone which is being decomposed and which intervenes, separating the healthy structure from the softened decay, is thin and much harder to be cut than that which we find in the white variety. This should always be removed; if left, the teeth around the plug will soon become discolored, and if moisture gets in, will soon soften and render the operation useless.

The white variety presents some singular characteristics, differing entirely from either the preceding. We here generally have extreme sensibility of the bone. This sensibility is not in the decomposed and already softened substance, but is in the part immediately beneath, and which is rapidly undergoing decomposition. I say rapidly because we generally find this form of disease makes rapid progress. May not this in part account for the extreme sensibility of the bone? This form of disease is also more frequently found in the young than the aged. There is therefore less density of bone, more vascularity, and hence we may look for more inflammation. The cartilaginous portion appears to be removed first, at least leaving a preponderance of the earthy matter as decayed substance. This is just the reverse from what is seen in the brown variety. The whiteness of the decay appears to be dependent upon the absence of animal matter, leaving the phosphate of lime in almost a pure state; mixed up it is true with foreign matter, which has lodged in the cavity. Yet when this is removed, we often find a white powdery substance, crumbling beneath the pressure of the cutting instrument, and are astonished with our patients to find the tooth so far gone.

This condition of decay has generally been considered the most difficult to treat. I have heard some dentists remark that "they did not believe that teeth thus effected could be saved." Let me ask why is this so, or rather is this the case? Is it not more attributable to an imperfect understanding of the disease? To the fact that many of our patients thus afflicted are young, rather ungovernable, &c. That indeed but few of our patients with such teeth will hold as firm as is desirable—we have a greater flow of saliva to contend against, we are anxious to give as little pain as possible, and may we not be deceived as it regards the extent to which the disease has penetrated the tooth. Is there not beneath the crumbly, powdery substance a layer of bone which should also be removed? I believe there is, and that unless this is cut away a few months will find a filling out, or that it is too small for the cavity.

This stratum of bone has so far been injured by the acute state of inflammatory action, that it must ultimately be dissolved. And may it not indeed have absorbed a sufficient amount of some solvent fluid, which must not only decompose this stratum; but as this is done, pass on to the healthy portion? One thing at least is certain, which is, that when this is removed the extreme sensibility of bone is also gone.

There is no variety of decay requiring such thorough work to arrest. I believe that an aching tooth will keep up the disease. It is important in all cases to remove from the mouth all teeth which are causing irritation, and cannot be preserved. Here it is absolutely necessary. But I am digressing somewhat from my subject. I thought it necessary, however, to allude to a few circumstances in connection with the different varieties of decay named, because these conditions should direct us in our operations, and determine the extent to which we should use our excavating instruments. I have not pretended to describe every condition of decay; for time would not permit. We have modifications of these forms of decay; causes exist which may at times blend two or all together. I have seen the white pass into the brown, and the brown to the black.

The cause of dental disease forms of itself a subject of vast importance to every dentist. We are now, however, only treating of one of the remedies. Enough has been said to give a tolerable idea of the manner of preparing the central cavities for a plug, and much has been here said, too, in relation to the disease which need not be repeated when we come to speak of the other class of cavities.

The cavity having been prepared for a filling, by the entire removal of the disease, the margin being firm, smooth, and somewhat regular, that is, not notched and ragged, or the edge frail and brittle. The walls extending inward, being as near perpendicular as possible, or if beveling, so as to slightly enlarge the cavity within, yet not so as to form a neck, but the wall to present a continuous plane, so that a fold of the gold may rest against it smooth and straight its entire length.

Let me here draw attention to one circumstance, which if neglected, will ultimately show itself around the margin of the filling. It is this, that in shaping these cavities, that is in removing the carious portion. If particular care is not taken, the diseased bone just beneath the enamel will not be removed and we will have a neck shaped cavity, the neck of which will be filled by caries. The decay will generally be found full as large at the entrance of the cavity, immediately under the enamel as within. A portion of the enamel yet apparently unaffected by the disease laps over the decay—this in the brown and white variety alluded to, may be still unchanged in color; yet with a proper shaped excavater which can be thrown in under the enamel, it will be found soft or undergoing decomposition. When this is ascertained to be the case, it shows that the enamel has not been sufficiently removed. This diseased bone I generally remove first, and then trim the border to suit; or as is often the case cut and trim, until I find this part of the cavity perfected. Just so in the use of the drill; the first one used cannot, and generally should not be as large as the decay, but after using one which will pass in readily, cut the decay from under the enamel; then a larger drill, and repeat this till this part of the cavity is perfected. As I before remarked, however, the first step in the preparation of the cavity, is the breaking down of the enamel which covers the decay with the cutting instruments, and this is done to the extent necessary, thoroughly to expose the disease.

We pass on to the next step in this operation, and this would be the preparation of the gold; for this should be prepared and ready for insertion before the cavity is washed out and dried. I am aware that various plans are adopted to accomplish this object. Some cut the gold in strips of about one fourth of a sheet; then roll into a cylindrical form, adding strip after strip to this roll till enough is added to fill the cavity—then make a few folds at one end, sufficiently large to be retained in the cavity when put in, till other folds are made with the plugger and forced upon it, filling up by these repeated folds the entire cavity. Others take two or three sheets of gold, lay carefully together, then fold once or twice, so as to get some ten or twelve thicknesses of the foil, then cut in strips as wide as the cavity, leaving these strips all united, then roll up one end into a ball, sufficiently large to be retained in the cavity, then fold in, in regular and successive layers, as much as can be forced into the cavity. This plan when done by an expert operator, goes far to the accomplishment of all that is desired

Some difficulties, however, present in the proper introduction of the gold in this manner, let us take for inance a central cavity. You commence with a strip introducing the folded end first, then with your instrument draw up and force in another strip; let this be done with all the care imaginable, and all these strips will not be of one length; some will have been drawn intirely within the cavity, while others will project without. A few *misfolds* of this kind will occasion two things; first, the too rapid filling up of the cavity within for the border; and second, an irregularly compacted surface for the filling, rendering it more difficult to put on a fine finish. The same objections hold good as regards the first plan.

But this condition of things is far preferable to that which often takes place. The first fold or ball is forced into the bottom of the cavity, the next on top of this, and so on, filling up from the bottom to the top. If this plan is adopted, and it certainly is the easier so far as the filling up of the cavity is concerned, yet what is to hold the last fold of gold. True this may be worked into (more or less) that which is beneath, yet, if I mistake not, such fillings will generally be found to wear down pretty fast, and be far easier picked out than when the folds are compacted in the other way. Others again roll up small balls of gold, and force in one after another, begining at the bottom of the cavity. The great desideratum undoubtedly is to fill up the entire cavity, make the gold as solid as possible, impervious to air or moisture, and so compacted and held together, that one portion cannot be removed without the whole, unless by the aid of a cutting instrument.

That plan which will secure the introduction of the most gold, and best fill up the entire cavity, is certainly the best, and that which experience and long practice has made easiest to one, may be for the want of this almost impossible to another. Well do I remember the first filling I attempted to introduce with my foil cut into strips, and with foil such as recommended by one of our best dentists. This foil was rather hard yet adhesive, so that when folded it would stick together—when once introduced, it made a hard plug and bore a fine polish; yet after long and tedious labor, I tore my plug entirely out, and took up my cylindrical rolls. This to me then was *the* plan. But one failure did not discourage me, I tried other foil, and at length nearly threw my rolls aside. Notwithstanding I did this and prefered my gold in strips, yet the objections already alluded to determined me to try another plan, and I commenced this in the central cavities, for I found these especially in the second molares superior, also inferior, to be as difficult to fill neatly as any other, which was anything like as accessible. I commenced by taking my strips, such as I had been using, these I cut, say one-third or fourth longer than the depth of my cavity. I then carefully rolled these into blocks of various sizes, having one sufficiently large to retain its position in the cavity when introduced. The object being to place these perpendicularly in the cavity, and fill up from the farthest portion of the cavity forward; but to do this the ordinary plugging instruments would not answer. For a long time I used a couple of pair of plyers, so shaped at the point, as to resemble the ordinary sharp pointed plugging instruments used for these cavities. I have now perfected what I call a set of plugging plyers or forceps, consisting of five pair, to these I shall probably add one or two more. The five are made from patterns of instruments most used in general filling. When closed, they resemble at the point these instruments, being sharp at the point, and so bent as to enter almost any cavity accessible with any instrument. When closed, therefore, they can be used as the ordinary instrument; and are thus often used for compacting the gold as introduced; and so admirably do they answer for this purpose, that in using the strips for filling, I use also these forceps. They possess one advantage over the ordinary plugger, which is, that with them you can catch hold of the strip at any point desired, carry it direct to the bottom of the cavity, and thus secure the desired length of fold. The spring of these instrument is such as to permit them to be closed with slight pressure, yet such as to force the blades some half inch apart, when this pressure is entirely removed. They are about five or six inches long, this should be determined by the length of hand, &c. Let us now take as an illustration of the method of using these instruments, the filling of a central cavity in an inferior molar. The cavity has been prepared, a napkin is held in place around the tooth with the speculum. The cavity has been thoroughly dried by the use of cotton, tissue paper, lint, or some such substance, which will most readily take up the moisture. The foil has been rolled up in blocks to suit the size of the cavity requiring some four, six, eight, or ten, to fill the cavity depending on its size. These blocks you have laid perfectly within your reach. You then take a pair of forceps bent at near right angle; with these take first your largest block, place this on end in the posterior part of the cavity, having the cut border of gold, projecting out of the cavity, say one-fourth the depth of the cavity; the other end, of course, resting on the bottom. When this block is to its place, and forced back solid against the posterior wall of the cavity, another next in size is introduced in like manner, and forced back against this; but it must here be remembered that this block will not as the first reach across the cavity; hence it should be forced to one side. The third block to the other, the fourth to the center, and so on until the cavity is completely filled. As each block is introduced it is pressed solidly back to its place, the last block introduced has a portion of the strip unrolled, and as much of this as *can be*, is forced in as a closing wedge. We here see the propriety of having the walls of the cavity perpendicular, so that these blocks will rest solid against them without being bent or broken down. In large cavities, after the introduction of each, or two or three blocks, a strong plugging instrument is used to force back and down these blocks, and thus give room for the introduction of others. We have here a filling made up of continuous layers of gold, each layer projecting above the cavity. No fold too long or too short; for in deep and large cavities the bottom can and should be first filled up to a level with the shallowest part of the cavity, by the introduction of one or more blocks laid in on their sides, and made perfectly solid. And this I do in all those cavities of much size, and where there is much tenderness at the bottom. A little experience will soon direct the most proper size and firmness of the blocks to be used, also their length. I have found that if properly introduced, and consolidated as introduced, that but little pressure on their ends is necessary to finish the compacting of the gold; yet this part of the operation is very important; as pressure should be made as the tooth will well bear. For this part of the operation a few strong instruments are necessary. These I have constructed in a manner which render them fit only for this purpose. I take the ordinary straight, curved, and flat compressing instruments, but the points are cut like a file, the sides of the flat are also made this way. These are to be used after the introduction of the gold. Two objects are had in view in having my compressing instruments thus made. The first is, they can be held more readily to the place; not being half so liable to slip. The second, they compact and unite the surface, and by a rotary or sliding motion, file off as it were, that portion of the gold which cannot be forced into the cavity. In the use of these instruments a continuous, straight, heavy pressure is not the most effectual; but the motion should be made so as to rock the instrument, as it were, back and forward on the gold.

Fillings inserted in the manner just described, in central cavities, where these have also been properly prepared, will not wear out by any process of mastication, short of that which will wear the teeth away.

The manner of filling all the central cavities is so near the same that a separate description is not necessary. I shall therefore only allude to a few particulars, which change to a certain extent the manner of introducing gold. Occasionally we have a cavity too small for entire block filling. When this is the case, I use a small block rolled up on the end of a strip of gold, and force this into its proper place; then, with a sharp pointed plugger, carry in layer after layer until the cavity is well filled. It is well to have at all times a small strong spear-pointed plugger, to introduce the last folds of the gold. An instrument well adapted for the commencement of a filling, will not always be at all suitable to finish with.

I have alluded to one form of central cavity, which requires at this time a passing notice. This is where the sutures or fissures crossing the molar teeth, particularly the inferior, is the seat of disease; and this follows these sutures to their extremes. But here they dip somewhat deeper than in their entire course; and the disease has here made progress somewhat in proportion to that at the center. This condition of things will often make one pretty large cavity in the center, with two, three, or four smaller ones running into this, here taking an inferior molar for example, I should fill the posterior first, second the inner, third the outer, then the center, finishing on the anterior. I wish always to fill that portion of the cavity farthest from me first, because in this way I retain a perfect view of that portion yet unfilled, and can see that my blocks or folds of gold are properly arranged.

I have not alluded to other instruments than the speculum for holding the tongue in place, and keeping the tooth dry, because I have found none which would compare to this for general use. The corner of a napkin neatly folded, and thrown around the posterior molar or dens sapientia, so that the end shall be on the outside against the cheek, the larger fold then is over the tongue. A pledget or two of cotton may still be forced in around the tooth to be filled, this will absorb any saliva, which might otherwise reach the cavity. This, however, is done after the speculum is placed over the napkin. A description of this instrument is unnecessary, for I believe it is generally used. It is held in place by the patient, and if properly constructed, and placed in the mouth so as to produce no pain when pressed upon, is of incalculable service. It is, however only applicable to the teeth of the inferior maxillae. The same difficulty is not generally experienced in keeping the superior teeth free from moisture, during the operation of filling, yet even here we sometimes meet with difficulty.

Some place a large piece of cotton on the labial surface of the teeth; hold this in place with the fore-finger of the left hand; with the thumb resting against the palital surface of the tooth; or reverse this order of thing when applied to the teeth of the right side. Others use a cheek holder made of pearl. This at times may answer a good purpose, yet it keeps the left had too far from the teeth, so much so that it can be of no service in handling the gold. The corner of a napkin used as the piece of cotton, I have generally found sufficient, and full as convenient. Custom in this as well as in almost every thing, will generally give success and choice of the method used. In the upper teeth, as we advance back in the mouth, difficulty of access and consequently of filling increases. The manner of preparing the gold, of introducing and compressing, has been described. This is applicable to the use of gold; also tin when used in blocks. But I have generally found this metal in foil to possess so little adhesiveness, that the folds will not well adhere together; hence when using this article other than in blocks, I have cut in broad strips and rolled in cylindrical form. Of late years, however, it is so little used that I have taken no special pains to test the best method of using it, unless, indeed, a few large fillings inserted in the form of blocks; these soon acquired a solidity of surface, and bore a better finish than I have usually expected from this metal. No amount of labor, however; can make it as hard as gold. After the filling has been introduced and consolidated, the projecting foil must be cut to the proper level, and thus prepared for a proper finish. This is done with files and scraping instruments, so constructed as to accomplish this object with very little trouble. The perfection to which files have been made for this purpose, the warmest gratitude of the profession is due Mr. Murphy, the manufacturer, as well as those of our brethren, who have taken the pains to form, and labor to supply him with the requisite patterns. With these instruments the plug is cut down level with the margin of the cavity; then with slightly blunted pluggers, pressure is made around the center border of the filling. The edges trimmed of all roughness. The plug must be trimmed so that the antagonizing tooth will not strike upon it.

---

Note.—The address on filling teeth will be continued in our next number. Time did not permit a full and complete discussion of this subject. The treatment of sensitive or inflamed bone preparatory to filling; the capping of the nerve, also the killing; with the method of filling thereafter, was not dwelt upon. It was requested that I should carry out the subject in full, and publish in the Register. This, if permitted, will be done; but it will consume more space than can be appropriated in one, or perhaps two additional issues.

